# Solid State Characterization and Dissolution Enhancement of Nevirapine Cocrystals

**DOI:** 10.34172/apb.2021.087

**Published:** 2020-09-22

**Authors:** Prabhakar Panzade, Giridhar Shendarkar, Deepak Kulkarni, Santosh Shelke

**Affiliations:** ^1^Department of Pharmaceutics, Srinath College of Pharmacy, Aurangabad, India.; ^2^Nanded Pharmacy College (poly), Nanded, India.

**Keywords:** Cocrystal, Nevirapine, Surfactant, pH, Dissolution

## Abstract

***Purpose:*** Novel cocrystals of nevirapine (NP) were designed and prepared with salicylamide and 3-hydroxy benzoic acid (3-HBA).

***Methods:*** The cocrystals were prepared by solvent drop grinding method by adding few drops of acetone to enhance the solubility and dissolution. The drug and cocrystals were characterized by differential scanning calorimetry (DSC) and powder x-ray diffraction (PXRD). The solubility of NP, its wet ground form, and cocrystals were investigated at different pH. Moreover, the effect of surfactant on solubility of cocrystals was also studied. Finally, intrinsic dissolution rate (IDR) and stability of cocrystals was examined.

***Results:*** The characterization of cocrystals by DSC and PXRD revealed formation of new solid forms due to changes in thermogram and PXRD pattern. The cocrystal of NP with 3-HBA showed 4.5 folds greater solubility in pH 1.2 buffer and 5.5 folds in 1% Tween 80 as compared to original drug. IDR of cocrystals was higher than the pure drug in 0.1 N hydrochloric acid (HCl). Moreover, cocrystals were found physically stable after 3 months as evident from unchanged IDR.

***Conclusion:*** Hence, the present research indicates the new stable solid forms of NP with improved dissolution rate than pure drug.

## Introduction

The significance of solubility and dissolution rate of drugs has been explicitly understood for the *in vivo* performance of the drug and/or drug product.^1^ Several approaches have been used to improve solubility and dissolution like micronization, solid dispersion, solubilisation, etc.^2^ However, pharmaceutical cocrystals have attracted enormous attention from the pharmaceutical industry owing to commercial potential and ability to modulate solubility, dissolution, stability, pharmacokinetics, etc. of drugs. Further, the entry of the Food and Drug Administration (FDA) approved cocrystal products in the market and their presence in clinical trials pipeline provided an impetus to cocrystal research in the academia and pharmaceutical industry.^3,4^ Moreover, it provides an opportunity to industry for filing patents related to new solid forms and launches old drugs in new forms extending the life cycle. The potential of cocrystals to modulate solubility is the biggest benefit as it is indispensable for the performance of the drug *in vivo*. Cocrystallization has been endorsed as an approach to tailor the solubility and/or dissolution rate of drugs. The most widely accepted definition of cocrystal is ‘cocrystals are solids that are crystalline single-phase materials composed of two or more different molecular and/or ionic compounds generally in a stoichiometric ratio which are neither solvates nor simple salts’.^5^ The chief hypothesis cited in the literature for enhanced solubility and dissolution is the altered structure of the drug and weak bonds involved in the cocrystal.^6^

**Scheme 1:**
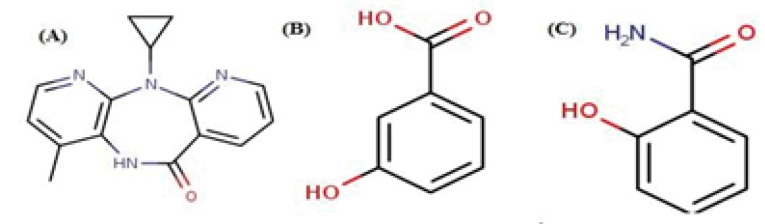
Structure of nevirapine (A), 3-Hydroxy benzoic acid (B), Salicylamide (C).

Nevirapine (NP) is a BCS class-II non-nucleoside reverse transcriptase drug having inadequate aqueous solubility of 0.1 mg/mL and high permeability (Log P 2.5). NP (pKa 2.8), at higher doses exhibit solubility limited absorption with low bioavailability.^7,8^ It is possible to prepare novel solid forms of NP with greater therapeutic efficacy and commercial value via cocrystallization. The various cocrystals of NP with amides, carboxylic acid, amino acids have been reported possessing enhanced solubility and dissolution.^9^ However, cocrystals of NP with salicylamide and 3-hydroxy benzoic acid (3-HBA) have not been reported till date. Besides, NP contains an amide group which could be the probable site for the preparation of cocrystals with selected coformers. Scheme 1 shows the structure of NP and coformers. Moreover, H-bonding functionalities in the NP could form cocrystal through the supramolecular synthon approach.^10^ This research was undertaken to check the feasibility of amide/amide or amide-acid heterosynthons in the bicomponent system and to fine-tune the solubility and dissolution rate of NP through cocrystal approach using green methods along with the impact of pH and surfactants on cocrystal solubility.

## Materials and Methods

NP was received as gift sample from Mylan laboratories limited, India. The coformers and other chemicals were purchased from Sigma Aldrich, Mumbai, India and used without further purification.

### Preparation of cocrystals

Solvent drop grinding method was used to prepare cocrystals. An Equimolar amount of NP and coformers (salicylamide and 3-HBA) were ground in mortar and pestle for 30-45 minutes by adding few drops of acetone. The resulting product was dried overnight and stored in air tight container until physicochemical characterization. NP was similarly treated with acetone to serve as a positive control.^11^

### Cocrystal characterization

#### Differential scanning calorimetry (DSC)

The thermal behaviour of the pure NP and potential cocrystals was examined. This was accomplished using a DTA-50 module differential thermal analyzer (Schimadzu, Japan). The dry sample (3 mg) was loaded into aluminium pan which was crimped and the sample was heated at a rate of 10°C/min covering the temperature range of 10 to 300°C using a steady flow of nitrogen. TA-60WS thermal analysis workstation and software was used for recording the data.

#### Powder X-ray diffraction (PXRD)

NP and potential cocrystals were analyzed by PXRD to identify new crystalline phase. PXRD patterns of NP and processed NP and coformer samples were recorded using a Brucker X-ray diffractometer (Madison, WI, USA) organized with an online recorder. The data including 2θ value was acquired by D8 adjust software linked to the instrument.

### Solubility analysis

The equilibrium solubility of NP, treated NP and cocrystals was determined in distilled water. After equilibrium, samples were filtered through a membrane filter (0.45 μm) and analyzed using UV spectrophotometer (Shimadzu 1800) at λmax of 313 nm.

Similarly, pH dependant solubility of cocrystals and influence of various surfactants like Tween-80, on cocrystal solubility was also investigated.^12^

### Intrinsic dissolution rate (IDR)

IDR measurement was conducted for NP and its cocrystals in 0.1 N HCl using USP type II dissolution apparatus (Electrolab, Mumbai) at 37±0.5 °C and stirring rate 100 RPM. The surface area of the compressed pellet was kept constant and IDR was calculated as the cumulative drug release per unit time per unit exposed surface area of the pellet.^13^

### Physical stability

Cocrystal products were exposed to a constant temperature and humidity conditions for 90 days to assess the stability at 25 °C and 60% RH. The alterations in IDR were used as criterion to examine physical stability.^14^

## Results and Discussion

### DSC

**Figure 1:**
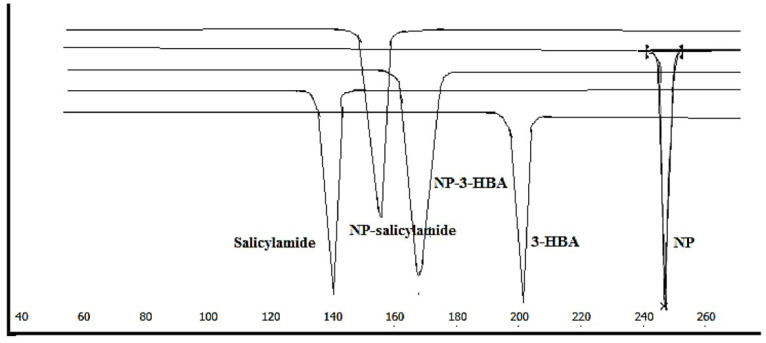
DSC thermograms of NP (nevirapine), 3-HBA (3-hydroxybenzoic acid), Salicylamide, NP-3-HBA (nevirapine-3-hydroxy benzoic acid cocrystal), NP-salicylamide (nevirapine-salicylamide cocrystal).

The DSC thermogram shows a single endothermic event at 245°C ascribed to the melting point of the drug. The melting points of the coformers were obtained at 141.2°C and 205°C respectively for salicylamide and 3-HBA. The observed melting behaviour was almost the same as reported in the previous research.^9^ The thermal behaviour of the new crystalline solid form was distinct than the drug and coformers. The melting of NP-salicylamide cocrystal (NP-S) and nevirapine-3-hydroxybenzoic acid cocrystal (NP-3-HBA) was observed at 157.6°C and 171°C respectively (Figure 1). The melting points of both the solid forms were in between drug and coformers. Such behaviour has been reported for cocrystals of bicalutamide. The change in melting behaviour signals the formation of a new solid form.^15^ Melting point is affected by chemical structure, intermolecular interactions, molecular symmetry etc. Moreover, the change in meltingpoint as compared to starting components was taken as a basis for the identification of naproxen cocrystal. The lower melting cocrystals also indicate the higher dissolution.^16^

### PXRD analysis

**Figure 2:**
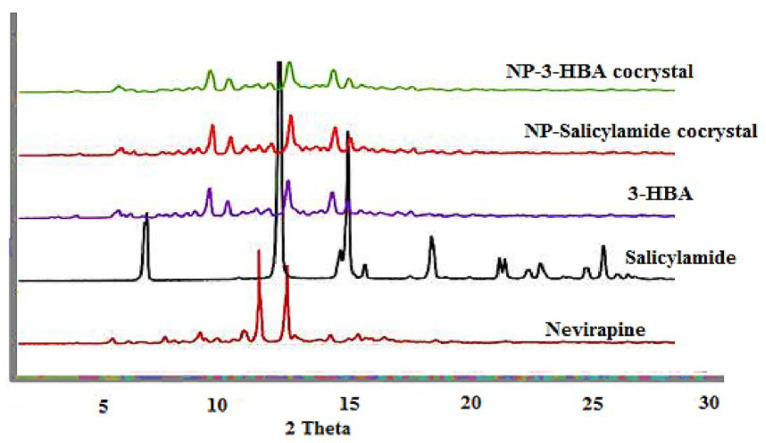
PXRD patterns for NP (nevirapine), Salicylamide, 3-HBA (3-hydroxybenzoic acid), NP-salicylamide (nevirapine-salicylamide cocrystal), NP-3-HBA (nevirapine-3-hydroxy benzoic acid cocrystal).

The PXRD pattern of pure drug, coformers and two cocrystals is presented in Figure 2. The principle diffraction peaks corresponding to NP were observed at 2θ values of 9.1, 13.4, 14.6, 19.3, 23.4, 23.9, 26.3 degree. The coformers exhibited characteristic diffraction peaks at distinct 2θ values and similar results were reported in the previous investigation. The diffraction peaks for 3-HBA and salicylamide are presented in Figure 2. The difference in PXRD pattern is used as a vital tool to ascertain cocrystal production. The changes in diffraction peaks were noted in cocrystals with a slight change in intensity. The appearance of distinct and some additional diffraction peaks as compared to drug and coformers suggest the formation of new crystalline solid form. The new peaks which were not present in drug and coformers were 3.2, 6.9, 7.4, 10.1, 18.2, 24.8 for NP-salicylamide cocrystal and 4.2, 4.9, 12.3, 14.9, 16.2, 23.5 for NP-3-HBA cocrystal respectively. Similar modifications in PXRD patterns of respective cocrystals from 5-fluorouracil and coformers were used to assess cocrystal formation.^17^

### Solubility analysis

**Figure 3:**
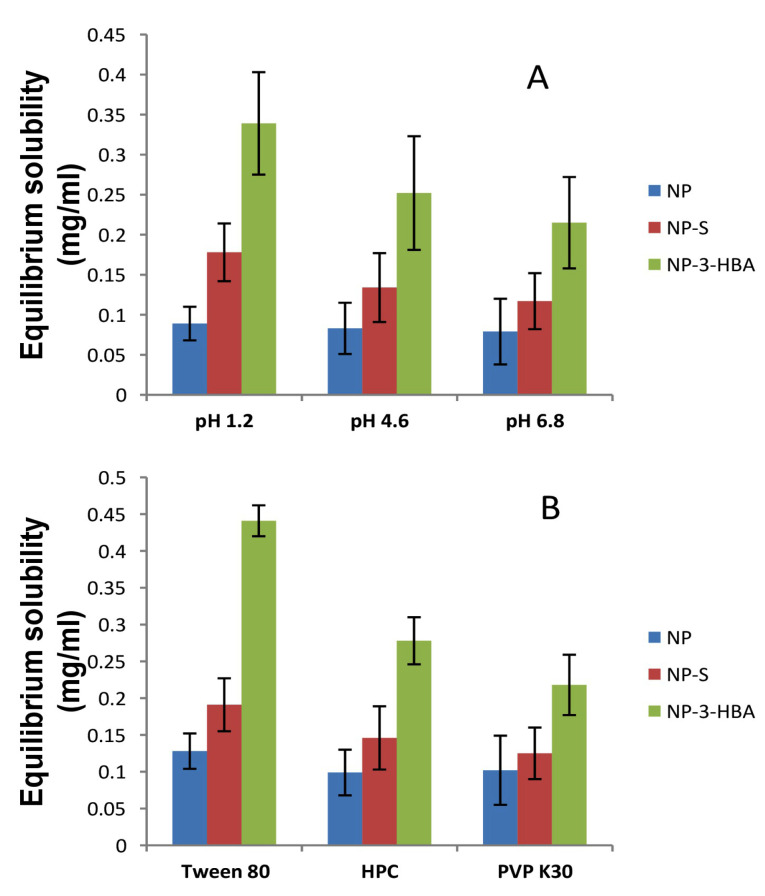
Effect of pH (A) and surfactant (B) on equilibrium solubility of NP (nevirapine), NP-S (nevirapine-salicylamide cocrystal), NP-3-HBA (nevirapine-3-hydroxy benzoic acid cocrystal).

The solubility of NP, cocrystals and wet ground drug was investigated in water and different pH buffers (Figure 3). The study was executed to check the behaviour of cocrystals in the physiological environment. The equilibrium solubility of the drug in water was 0.077 mg/ml indicating poorly water soluble drug (solubility < 0.1 mg/mL). The solubility of the wet ground drug was 0.082 mg/ml which rule out the possibility of solubility improvement due to simple physical mixture. The solubility of NP was pH dependant as greater solubility was obtained in pH 1.2 buffer. The NP-3-HBA cocrystal solubility was improved 4.5 folds (0.339 mg/mL) in pH 1.2 buffer. This is owing to weakly basic nature of drug which results in greater ionization and solubility at lower pH. The wet ground drug showed no change in solubility hence it was not considered further. Similarly, the effect of surfactant on cocrystal solubility was examined in pH 1.2 buffer. The NP-3-HBA cocrystal exhibited 5.5 folds increase in solubility (0.441 mg/mL) in Tween 80. The solubility of NP-salicylamide cocrystal was enhanced 2.5 folds as compared to a drug after addition of surfactant. The 1% Tween 80 has a substantial effect on cocrystal solubility in comparison to other surfactants. Interestingly, equilibrium solubility of both cocrystals altered due to the addition of surfactants. The proportion of drug-micelle mainly contributed to solubility. This may be due to the micellar solubilisation of drug in surfactant. Such results were obtained for the myricetin cocrystals.^18^ In addition, cocrystals of apremilast with nicotinamide, caffeine and acetylsalicylic acid exhibited an increase in solubility with the effect of pH and surfactant.^19^

### Intrinsic dissolution rate

**Figure 4:**
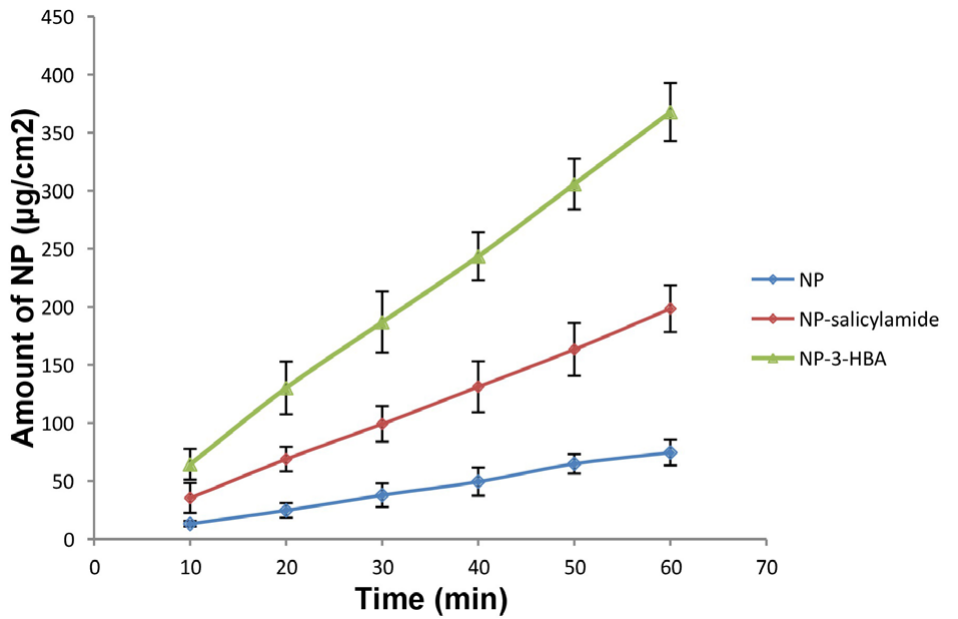
Dissolution profiles of NP-nevirapine, NP-S: nevirapine –salicylamide cocrystal, NP-3-HBA:nevirapine 3-hydroxy benzoic acid cocrystal.

In vivo dissolution rate of the drug could be strongly connected with IDR. Therefore, IDR of drug and cocrystals was determined in 0.1 N HCl (pH 1.2) by adding 1% Tween 80. The IDR profiles are presented in Figure 4. The IDR of cocrystals was augmented as compared to pure drug. The IDR of NP, NP-salicylamide cocrystal and NP-3-HBA cocrystal was found 74.58 μg cm^-1^min^-1^, 198.48 μg cm^-1^min^-1^, and 367.78 μg cm^-1^ min^-1^ respectively indicating the potential of cocrystals to improve the dissolution rate of NP. The increase in IDR can be attributed to high solubility, low melting point and weaker crystalline structure of cocrystals.^20^

### Physical stability

The physical stability of NP and its two cocrystals were investigated for three months by determining the IDR. However, both the cocrystals were stable at the conditions of the study as evident from the unchanged IDR profiles after storage. The IDR of NP-salicylamide and NP-3-HBA cocrystal was found 195.68 μg cm^-1^min^-1^ and 363.35 μg cm^-1^ min^-1^ respectively. The identical results were reported for the cocrystals of zaltoprofen with nicotinamide, and unchanged dissolution profile before and after stability study were used to ensure stability.^21^

## Conclusion

New cocrystals of NP with enhanced solubility, dissolution and physical stability using salicylamide and 3-HBA as coformers have been produced via solvent drop grinding method. The prepared cocrystals were confirmed by DSC and PXRD. The establishment of *in vitro-in vivo* correlation will provide further idea about the commercial potential of these cocrystals.
